# “Overdiagnosed or Misdiagnosed” – A Classic Case of Pseudohallucinations and Maladaptive Daydreaming When a Diagnosis of Adult ADHD Was Missed

**DOI:** 10.1192/bjo.2025.10734

**Published:** 2025-06-20

**Authors:** Saima Jehanzeb, Amna Javaid

**Affiliations:** 1 American Centre for Psychiatry and Neurology, Al Ain, Abu Dhabi, UAE; 2 Psychiatry UK Online, UK, United Kingdom

## Abstract

**Aims:** Background: Attention deficit hyperactivity disorder (ADHD) can present differently in adults and often goes misdiagnosed due to overlapping symptoms. Maladaptive daydreaming may also be misdiagnosed, as it shares similarities with dissociative symptoms, depersonalization, derealization, and temporal lobe epilepsy. Jasper has identified pseudohallucinations as akin to normal perception and occurring in the inner subjective space. Adults with ADHD often experience zoning out, daydreaming, and cognitive hyperactivity, which may resemble psychosis.

**Methods:** Case Report.

This case study highlights the relationship between pseudohallucinations, maladaptive daydreaming, and ADHD in adults. A 19-year-old patient with a childhood history of trauma and isolation presented with hallucinations that began at age eight and progressed over time. She reported hearing up to 50 voices, described as “good and friendly”.

The voices were connected to multiple families and included visual, tactile, and auditory modalities. She claimed she could touch, feel, and see them, identifying them as her friends. She sought guidance from these voices and communicated with most of them. The patient had good insight and understanding of her condition. She referred to these entities as imaginary beings that served as a coping mechanism for stress and loneliness. She experienced episodes of persistent zoning out, living in an imaginary world, and actively engaging in conversations with multiple internal voices. Depersonalization, derealization, and dissociation led to her isolation, adversely affecting her interactions with her real family and academic performance. She had been prescribed antidepressants to manage her anxiety and insomnia, but these were discontinued due to no response previously.

To rule out organic causes for her hallucinations, an MRI brain, EEG, and comprehensive tests were performed, which excluded epilepsy. An ADHD assessment was conducted using standardized tools and DSM–5 diagnostic criteria, leading to a diagnosis of ADHD combined type.

**Results:** After starting methylphenidate (Concerta 54 mg OD), the patient experienced significant resolution of symptoms, including improved focus and no daydreaming episodes resulting in the loss of internal voices. This sudden quietness caused distress and adjustment difficulties, triggering identity disruption due to her long-term reliance on these voices for emotional regulation. Dissociation and pseudohallucinations were reinterpreted as manifestations of cognitive inattention, emotional dysregulation, and an overactive inner monologue.

**Conclusion:** The internal voices were recognized as non-psychotic self-dialogue, a phenomenon observed in ADHD. Zoning out, daydreaming, and depersonalization were linked to severe attention deficit disorder. ADHD overlaps with dissociative symptoms, depersonalization, and pseudohallucinations. Understanding the diverse manifestations of ADHD is crucial for accurate diagnosis and effective management.

